# Access to High-Resolution Anoscopy Among Persons With HIV and Abnormal Anal Cytology Results

**DOI:** 10.1001/jamanetworkopen.2024.0068

**Published:** 2024-03-01

**Authors:** Sun Hee Rim, Mona Saraiya, Linda Beer, Yunfeng Tie, Xin Yuan, John Weiser

**Affiliations:** 1 Division of Cancer Prevention and Control, National Center for Chronic Disease Prevention and Health Promotion, Centers for Disease Control and Prevention (CDC), Atlanta, Georgia; 2 Division of HIV Prevention, National Center for HIV, Viral Hepatitis, STD, and TB Prevention, CDC, Atlanta, Georgia; 3 DLH Corporation, Atlanta, Georgia; 4 Now with Global Medical and Scientific Affairs, Merck Research Laboratories, North Wales, Pennsylvania

## Abstract

This cross-sectional study evaluates use and availability of follow-up anoscopy among persons at highest risk for anal cancer.

## Introduction

Persons with HIV (PWH) in the US are 28 times more likely to be diagnosed with anal cancer than persons without HIV.^1^ Risk is particularly high among men who have sex with men,^2^ transgender women,^3^ and PWH 45 years or older.^2^ Identifying and treating anal cancer precursors can reduce cancer incidence among PWH.^3^ For PWH with abnormal cytology results, follow-up high-resolution anoscopy (HRA) is recommended.^4^ We estimated the number of HRA-eligible PWH at highest risk for anal cancer lacking HRA access because it was not available through their HIV care facility.

## Methods

Data were from the 2019 Medical Monitoring Project (MMP), the only US population–based survey of PWH 18 years or older. We also analyzed a 2021 supplemental survey on HRA availability (on-site or by referral) at facilities where 2019 MMP participants received HIV care. Additional information on MMP methods is provided in eAppendix and eMethods in Supplement 1 and elsewhere.^5^ In accordance with the Common Rule, this cross-sectional study was exempt from ethics review because MMP is a public health surveillance activity. Participants provided oral or written informed consent. We followed the STROBE reporting guideline.

We estimated weighted population frequencies and percentages (with 95% CIs) of PWH eligible for HRA by risk group.^4^ These PWH at highest risk included (1) gay, bisexual, and other men who have sex with men (GBMSM) or transgender women 35 years or older^2,3^ and (2) other PWH 45 years or older.^2^ We examined abnormal anal cytology results (≥atypical squamous cells of undetermined significance [≥ASC-US]) of PWH at highest risk who had cytology during the past 2 years (scenario A). We applied the percentage with abnormal results to estimate the number with abnormal results if all PWH at highest risk had cytology (scenario B) and if all PWH at highest risk who lacked access to HRA on-site or by referral had cytology (scenario C). Analyses accounted for MMP’s complex sample design. Data were analyzed using SAS 9.4 (SAS Institute) and SUDAAN 11.0.3 (RTI International).

## Results

Overall, an estimated 31 200 PWH at highest risk for anal cancer (GBMSM or transgender women ≥35 years; other PWH ≥45 years) had abnormal anal cytology results (≥ASC-US) in 2019 (Table). If all PWH at highest risk had cytology and the same distribution of results as those who were actually tested, 422 888 (95% CI, 350 869-494 906) would have had abnormal results. Under the same assumptions, an estimated 124 386 (95% CI, 89 421-159 352) PWH with abnormal results would have no access to follow-up HRA through their HIV care facility.

**Table.** Estimated Number and Percentage of PWH at Highest Risk for Anal Cancer With Abnormal Cytology Results Who Would Not Have Access to HRA^a^

Cytology results for PWH at highest risk for anal cancer^b^	No. of MMP participants^c^	Weighted population frequency (95% CI)		Cytology result within each group, weighted % (95% CI)		PWH for whom HRA would be recommended, weighted population frequencies (95% CI)		
		PWH	PWH seen at facilities without HRA on-site or by referral	Including PWH without cytology	Excluding PWH without cytology^d^	Scenario A: PWH who had cytology	Scenario B: if all PWH had cytology	Scenario C: if all PWH seen at facilities without HRA on-site or by referral had cytology
All PWH at highest risk^e^	3136	787 500 (715 124-859 876)	231 632 (175 754-287 509)	NA	NA	NA	NA	NA
Negative	119	NA	NA	3.4 (2.0-4.9)	46.3 (38.6-54.0)	NA	NA	NA
≥ASC-US	130	NA	NA	4.0 (2.6-5.4)	53.7 (46.0-61.4)	31 200 (18 509-43 891)	422 888 (350 869-494 906)	124 386 (89 421-159 352)
ASC-US	68	NA	NA	2.2 (1.3-3.0)	29.1 (23.3-34.9)	16 903 (9717-24 090)	229 163 (178 587-279 738)	67 405 (46 199-88 611)
LSIL	49	NA	NA	1.5 (1.0-2.0)	20.1 (13.4-26.8)	11 689 (6780-16 598)	158 288 (103 518-213 057)	46 558 (27 310-65 806)
HSIL, ASC-H, SCC	13	NA	NA	0.3 (0.0-0.7)^f^	4.5 (1.3-7.6)^f^	2608 (0-5243)	35 438 (10 419-60 456)	10 423 (2662-18 185)
No cytology	2861	NA	NA	92.6 (89.9-95.2)	NA	NA	NA	NA
GBMSM or transgender women aged ≥35 y	1656	426 975 (365 153-488 796)	122 259 (90 832-153 687)	NA	NA	NA	NA	NA
Negative	88	NA	NA	5.0 (3.0-6.9)	46.3 (38.5-54.1)	NA	NA	NA
≥ASC-US	102	NA	NA	5.8 (3.7-7.9)	53.7 (45.9-61.5)	24 308 (13 259-35 358)	229 286 (182 133-276 439)	65 653 (46 215-85 091)
ASC-US	52	NA	NA	2.9 (1.9-4.0)	27.2 (21.4-33.0)	12 325 (6990-17 661)	116 137 (86 058-146 217)	33 254 (22 089-44 420)
LSIL	39	NA	NA	2.3 (1.3-3.2)	21.0 (13.5-28.4)	9489 (4669-14 310)	89 665 (55 100-124 230)	25 674 (14 331-37 018)
HSIL, ASC-H, SCC	11	NA	NA	0.6 (0.0-1.2)^f^	5.5 (1.6-9.4)^f^	2494 (0-5124)	23 484 (6433-40 535)	6724 (1611-11 837)
No cytology	1445	NA	NA	89.2 (85.6-92.9)	NA	NA	NA	NA
All other PWH aged ≥45 y^g^	1480	360 525 (314 211-406 840)	109 372 (80 268-138 476)	NA	NA	NA	NA	NA
Negative	31	NA	NA	1.7 (0.5-2.8)^f^	46.3 (29.0-63.5)	NA	NA	NA
≥ASC-US	28	NA	NA	1.9 (0.9-3.0)	53.7 (36.5-71.0)	6891 (3412-10 371)	193 602 (126 593-260 611)	58 733 (34 124-83 342)
ASC-US	16	NA	NA	1.3 (0.4-2.2)^f^	35.7 (18.3-53.1)	4578 (1498-7658)	128 707 (63 551-193 863)	39 046 (17 168-60 924)
LSIL	10	NA	NA	0.6 (0.2-1.0)^f^	17.2 (6.3-28.0)^f^	2200 (730-3669)	62 010 (21 841-102 180)	18 812 (5784-31 840)
HSIL, ASC-H, SCC	2	NA	NA	0.0 (0.0-0.1)^f^	0.9 (0.0-2.2)^f^	114 (0-271)	3 245 (0-7942)	984 (0-2438)
No cytology	1416	NA	NA	96.4 (94.5-98.3)	NA	NA	NA	NA

Abbreviations: ASC-H, atypical squamous cells cannot rule out HSIL; ASC-US, atypical squamous cells of undetermined significance; GBMSM, gay, bisexual, and other men who have sex with men; HRA, high-resolution anoscopy; HSIL, high-grade squamous intraepithelial lesion; LSIL, low-grade squamous intraepithelial lesion; MMP, Medical Monitoring Project; NA, not applicable; PWH, persons with HIV; SCC, squamous cell carcinoma.

^a^ Data were collected during the 2019 MMP cycle and from a 2021 survey on HIV care facilities involving 2019 MMP participants.

^b^ Anal cytology results included negative, ASC-US, LSIL, HSIL, ASC-H, and SCC. The at least ASC-US category represents the sum of all results, excluding negative results. Categorization of PWH at highest risk was based on age, gender identity, sexual orientation, and anal cytology results.

^c^ Cytology results may not sum to the total for the respective risk group due to missing data in medical records abstraction.

^d^ The sum of at least ASC-US and negative cytology results totals 100%.

^e^ All PWH at highest risk included GBMSM or transgender women 35 years or older and all other PWH 45 years or older.

^f^ Coefficient of variation was 0.3 or greater. The estimate may be unstable and should be interpreted with caution.

^g^ The all other PWH 45 years or older group excluded GBMSM and transgender women 35 years or older.

## Discussion

National guidelines recommend that anal cytology should not be performed without the ability to refer for HRA,^4^ but we found that a substantial number of PWH at highest risk of anal cancer may not have access to HRA on-site or by referral at their HIV care facility. With anal cancer prevention by diagnosis and treatment of cancer precursors now demonstrated,^3^ improvements in screening and early detection, including systematic monitoring of such efforts, are needed. Improvements may include expanded HRA training or certification, modified patient care workflow to encourage clinicians to perform or refer for screening, and increased education and awareness about symptoms among patients and clinicians. Anal cancer disproportionately affects subpopulations in regions with lower socioeconomic status.^6^ If screening or prevention improvements are not equitably implemented, they can increase rather than reduce disparities.

Study limitations include HRA availability data reported by facility staff, which might be subject to information bias. Additionally, some participants might have had cytology at facilities other than those we analyzed; however, less than 10% of participants reported receiving HIV care at more than 1 facility. Finally, cytology results among all PWH at highest risk may differ from those who were actually tested.

This study’s nationally representative estimates can inform large-scale implementation of anal cancer prevention efforts. Given the limited supply of clinicians who can perform HRAs and the number of potentially HRA-eligible persons, HRA capacity may be the rate-limiting factor in precancer treatment among PWH.

## Notes

Supplement 1. **eAppendix.** Ethical Consideration and Informed Consent of Study Participants **eMethods.** Study Sample and Analysis

Supplement 2.
Data Sharing Statement
