# Supplementary material for: Access to High-Resolution Anoscopy Among Persons With HIV and Abnormal Anal Cytology Results
Source: JAMA Netw Open. 2024 Mar 1;7(3):e240068. doi: 10.1001/jamanetworkopen.2024.0068 (PMC10907917; doi:10.1001/jamanetworkopen.2024.0068)
Supplement: Supplement 1. — eAppendix. Ethical Consideration and Informed Consent of Study Participants eMethods. Study Sample and Analysis [file jamanetwopen-e240068-s001.pdf]

Supplemental Online Content

Rim SH, Saraiya M, Beer L, Tie Y, Yuan X, Weiser J. Access to high-resolution anoscopy among persons with HIV and abnormal anal cytology results.
JAMA Netw Open. 2024;7(3):e240068. doi:10.1001/jamanetworkopen.2024.0068

eAppendix. Ethical Consideration and Informed Consent of Study Participants

eMethods. Study Sample and Analysis

This supplemental material has been provided by the authors to give readers additional information about their work.

© 2024 Rim SH et al. *JAMA Network Open.*

eAppendix. Ethical Consideration and Informed Consent of Study Participants

MMP is a public health surveillance activity and thus institutional review board approval is not required. Oral or written informed consent was obtained from all participants. For the MMP facility survey, given that respondents were facilities and no information was collected on individuals, human subjects' approvals were not required.

© 2024 Rim SH et al. *JAMA Network Open.*

eMethods. Study Sample and Analysis

The Medical Monitoring Project (MMP) is a complex sample survey of US adults with diagnosed HIV that produces nationally representative estimates of behavioral and clinical factors. It is sponsored by the Centers for Disease Control and Prevention.

MMP uses a two-stage sampling design: (1) one-time sampling of 16 US states and Puerto Rico (from all US states, the District of Columbia, and Puerto Rico); and (2) annual sampling of adults aged ≥18 years with diagnosed HIV from the National HIV Surveillance System (NHSS), a US census of persons with HIV (PWH).

For the 2019 Medical Monitoring Project (MMP) data collection cycle, data collection occurred during June 1, 2019–May 31, 2020 via in-person or telephone interviews and medical record abstractions. In the 2019 cycle, participation was 100% at the state/jurisdiction level. In total, 9,700 persons were sampled from NHSS and 4,100 participated.

The population of inference for MMP is adults with diagnosed HIV (aged ≥18 years) living in the United States, the District of Columbia, and Puerto Rico. Details about MMP methodology, including sampling and weighting procedures are provided in the HIV Surveillance Special Report #28 Technical Notes (Centers for Disease Control and Prevention. Behavioral and clinical characteristics of persons with diagnosed HIV infection—Medical Monitoring Project, United States, 2019 Cycle (June 2019–May 2020). 2021. HIV Surveillance Special Report 28. https://www.cdc.gov/hiv/library/reports/hiv-surveillance.html.) The following references also provide more details about MMP methodology.

> Beer L, Johnson CH, Fagan JL, et al. A National Behavioral and Clinical Surveillance System of Adults With Diagnosed HIV (The Medical Monitoring Project): Protocol for an Annual Cross-Sectional Interview and Medical Record Abstraction Survey. *JMIR Res Protoc*. 2019;8(11):e15453. Published 2019 Nov 18. doi:10.2196/15453
>
> Johnson CH, Beer L, Harding RL, et al. Changes to the sample design and weighting methods of a public health surveillance system to also include persons not receiving HIV medical care. *PLoS One*. 2020;15(12):e0243351. Published 2020 Dec 3. doi:10.1371/journal.pone.0243351

Briefly, data were weighted based on known probabilities of selection at the state or jurisdiction level and person level, adjusted for multiplicity and nonresponse, and post-stratified to population totals from NHSS by sex, age, and race/ethnicity. The weighted data produced by MMP thus represents all adults with diagnosed HIV infection living in the United States.

We also analyzed data from the MMP facility survey, a supplemental survey of all HIV care facilities where 2019 cycle MMP participants received HIV care and had a medical record abstraction completed, to ascertain the availability of high-resolution anoscopy (HRA) on-site or by referral. Facility survey data were collected during July–November 2021. Details about the facilities survey are reported in Beer L, Williams D, Tie Y, McManus T, Yuan AX, Crim SM, Demeke HB, Creel D, Blackwell AD, Craw JA, Weiser J. The Capacity of HIV Care Facilities to

© 2024 Rim SH et al. *JAMA Network Open.*

Implement Strategies Recommended by the Ending the HIV Epidemic Initiative: The Medical Monitoring Project Facility Survey. J Acquir Immune Defic Syndr. 2023 Dec 1;94(4):290-300. doi: 10.1097/QAI.0000000000003290.

Briefly, for the MMP facility survey, 1,023 facilities were recruited to participate and 455 responded to the full survey. Because respondents were facilities and no information was collected on individuals, human subjects' approvals were not obtained. To generate person-level estimates of availability of HRA at the person's HIV care facility, we imputed missing facility data using 2 steps: recursive partitioning (trees) to create imputation classes and weighted sequential hot deck to produce imputed values. This ensured that person-level facility estimates had no missing values and minimized potential nonresponse bias.

Using 2019 MMP participant data, we estimated population frequencies, percentages and 95% confidence intervals (95% CI) of PWH aged ≥18 years and PWH with anal cytology during the past 2 years among all persons (N=4100), stratified by anal cytology results and availability of HRA at the person's HIV care facility based on the MMP facility survey. Missing data were excluded.

Anal cytology results are reported as negative, atypical squamous cells of undetermined significance (ASC-US), low-grade squamous intraepithelial lesion (LSIL), high-grade squamous intraepithelial lesion (HSIL), atypical squamous cells, cannot rule out HSIL (ASC-H), squamous cell carcinoma (SCC), and ≥ASC-US. The number with ≥ASC-US is the sum of ASC-US, LSIL, HSIL, ASC-H, and SCC.

For this analysis, we defined PWH at highest risk to be inclusive of: 1) GBMSM (defined as cisgender men who reported having sex with other cisgender men in the past 12 months or that their sexual orientation was gay or bisexual) or transgender women aged ≥35 years and 2) all other PWH aged ≥45 years.

In our results (table), we report 1) the number of MMP participants (unweighted) at highest risk for anal cancer by cytology results (column 2); 2) weighted population frequencies and 95% CI of PWH (column 3); 3) weighted population frequencies and 95% CI of PWH seen at HIV care at facilities without HRA access onsite or by referral (column 4).

In columns 7, 8, 9 of the table, we report the weighted population frequencies (and 95% CI) of PWH at highest risk for anal cancer for whom HRA would be recommended under three scenarios:

a) Based on actual anal cytology testing during the past 2 years (column 7)
b) If all PWH at highest risk had cytology (column 8)
c) If all PWH at highest risk who were seen at facilities not known to provide HRA onsite or by referral had cytology (column 9)

© 2024 Rim SH et al. *JAMA Network Open.*
