# Supplementary material for: Access to High-Resolution Anoscopy Among Persons With HIV and Abnormal Anal Cytology Results
Source: JAMA Netw Open. 2024 Mar 1;7(3):e240068. doi: 10.1001/jamanetworkopen.2024.0068 (PMC10907917; doi:10.1001/jamanetworkopen.2024.0068)
Supplement: Supplement 2. — Data Sharing Statement [file jamanetwopen-e240068-s002.pdf]

Data Sharing Statement

Rim. Access to High-Resolution Anoscopy Among Persons With HIV and Abnormal Anal Cytology Results. *JAMA Netw Open*. Published March 01, 2024.
doi:10.1001/jamanetworkopen.2024.0068

Data
Data available: No

Additional Information
Explanation for why data not available: Data Sharing Statement: Medical Monitoring Project (MMP) data are not available to be publicly shared due to security and confidentiality guidelines for the release of HIV surveillance data. However, CDC may provide on-site access to all relevant MMP data for researchers with approved analysis proposals who complete CDC Security and Confidentiality training. Proposals are reviewed and are prioritized based on their importance for public health, their scientific merit, and the needs and current workload of CDC staff. Inquiries should be made to Jason Craw (emf4@cdc.gov). All other project materials—including protocols and data collection instruments—are available to the public on the MMP website (https://www.cdc.gov/hiv/statistics/systems/mmp/index.html).
